# A multi-scale framework for BOD_5_ prediction from water quality to hydro-geomorphic interpolation

**DOI:** 10.1016/j.isci.2025.114177

**Published:** 2025-11-21

**Authors:** Amin Arzhangi, Sadegh Partani

**Affiliations:** 1 Faculty of Engineering, Civil Engineering Department, University of Bojnord, Bojnord, Northern Khorasan, Iran

**Keywords:** water geochemistry, environmental science, applied sciences

## Abstract

This study develops and validates a hydro-geomorphic interpolation model (HyRIM) to predict biochemical oxygen demand over 5 days (BOD_5_) concentrations at unmonitored locations and help optimize monitoring networks. The research first establishes the significant spatiotemporal heterogeneity of BOD_5_ drivers in the Haraz River Basin, Iran, revealing a distinct regime shift where physical parameters control BOD_5_ in the headwaters, while nitrate becomes dominant downstream and during colder seasons. The HyRIM integrates key hydro-geomorphic parameters, including stream order, channel sinuosity, and in-stream travel time, with hyperparameters optimized through a data-driven calibration process. Evaluated using a robust leave-one-out cross-validation (LOOCV) scheme, the final calibrated HyRIM demonstrates exceptional predictive power (R^2^ = 0.941, root-mean-squared error [RMSE] = 2.06), significantly outperforming conventional regression models. The findings demonstrate that HyRIM is a powerful tool for generating high-resolution pollution maps and designing more efficient monitoring strategies in complex river systems.

## Highlights

• BOD_5_ drivers shift from physical parameters to nitrate along the river continuum
• A hydro-geomorphic interpolation model (HyRIM) is developed and calibrated
• HyRIM achieves high predictive accuracy (CV R^2^ = 0.941) outperforming MLR models
• The framework enables monitoring network optimization and cost reduction

## Graphical abstract

**Figure:**
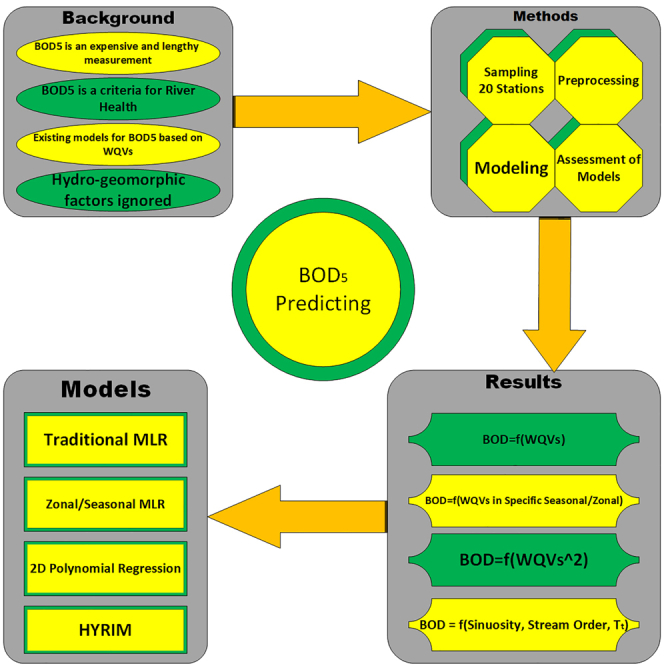

## Introduction

The quality of river water is critical to maintaining ecosystem health,^1^ and its degradation can lead to significant environmental problems, including the spread of diseases in humans and animals,^2^ loss of biodiversity,^3^ destruction of vegetation, and regional ecological imbalances.^4^ Pollution in river systems can release from both point and non-point sources,^5^ with human activities such as industrial discharges,^6^ agricultural runoff,^7^ and untreated wastewater^8^ contributing significantly to contamination. Additionally, certain natural processes, such as the over-reproduction of specific species, can also introduce substantial pollutants into surface waters.^9^

A key parameter in water quality monitoring is biological oxygen demand over 5 days (BOD_5_),^8^ which serves as an important indicator of organic pollution, oxygen depletion, and potential ecological changes in aquatic ecosystems.^10^ Despite its importance, BOD_5_ measurement is time-consuming and expensive,^11^ making it impractical for widespread or frequent monitoring. As a result, numerous studies have sought to develop alternative methods and predictive models based on other water quality variables (WQVs) that are easier to measure, faster, and more cost-effective, while still providing reliable assessments of water quality.^12^^,^^13^^,^^14^^,^^15^

The Streeter-Phelps (1920) model^16^ is a classic mathematical approach used to predict the dynamics of dissolved oxygen (DO) and BOD_5_ in natural water bodies like rivers. Additionally, several studies have predicted BOD_5_ based on WQVs,^17^^,^^18^^,^^19^^,^^20^^,^^21^ with variations primarily in the methods used, such as classical statistics and machine learning techniques.

Najafzadeh and Ghaemi^19^ used employed multivariate adaptive regression spline (MARS) and least square-support vector machine (LS-SVM) models to predict BOD_5_ and chemical oxygen demand (COD) using 200 field samples from the Karoun River, incorporating water quality indicators like electrical conductivity (EC), pH, and turbidity. Their models outperformed traditional methods and showed strong prediction accuracy. MARS and LS-SVM were also more effective than other AI models like artificial neural network (ANN) and adaptive network based fuzzy inference system (ANFIS) in this study.

Ooi et al.^21^ developed a BOD_5_ prediction model using machine learning methods like random forest, support vector regression, and multilayer perceptron, optimized with a genetic algorithm and sequential feature selection. The multilayer perceptron model performed the best with an R^2^ of 0.77 and a 15% error margin. In a field study from Jiangsu Province, China, it showed a 6% improvement in prediction accuracy.

Mishra et al.^17^ reported that, due to the limitations of traditional methods, machine learning models have been explored for BOD_5_ prediction. This study evaluates the performance of the extreme gradient boosting (XGBoost) model in predicting BOD_5_ using water quality parameters from the river Ganga in India and compares it with adaptive boosting and ANN models. The results demonstrated that XGBoost outperformed the other models, achieving the lowest root-mean-squared error (RMSE) of 0.5184 and a prediction success rate of 79.85%, offering a promising approach for addressing water quality challenges in rivers like the Ganga.

Several studies have explored various models for predicting BOD_5_ in rivers, including stream water quality models like QUAL2K for Iraq’s Diyala and Tigris,^22^ and ANN for the Danube River. The GREEN+ model has been used to assess organic pollution across Europe.^23^ Machine learning algorithms like ANN, support vector machine, random forest (RF), and gradient boosting machines (GBM), along with hybrid models, have been applied to rivers like Bangladesh’s Buriganga.^18^ Despite differing methodologies, these studies consistently identify organic pollutants like COD, total suspended solids (TSS), and total dissolved solids (TDS) as key predictors of BOD_5_ levels.

Previous research has thoroughly examined BOD_5_ levels and other WQVs in various regional rivers, employing a range of methods to enhance model accuracy. However, most predictor models focus solely on WQVs as predictors, without considering other environmental and river-specific factors such as flow regime, river morphology, topography, and ecosystem characteristics. Additionally, while previous studies emphasize the precision of models,^24^ they often overlook the importance of the cost associated with using certain WQVs as predictors.

This research is divided into two main sections. The first focuses on using WQVs as predictors, aiming to reduce sampling costs by selecting parameters that are both cost-effective and commonly measured *in situ*, while simultaneously improving prediction accuracy. The goal is to leverage river basin morphology^25^ to develop more precise models tailored to the specific characteristics of each basin. The second section explores an alternative approach to predicting BOD_5_ by reducing the number of BOD_5_ sampling stations needed for monitoring. Instead, it predicts BOD_5_ values at unsampled stations using data from other sampling locations, incorporating river features such as meanders and stream orders.

## Materials and methods

**Figure 1:**
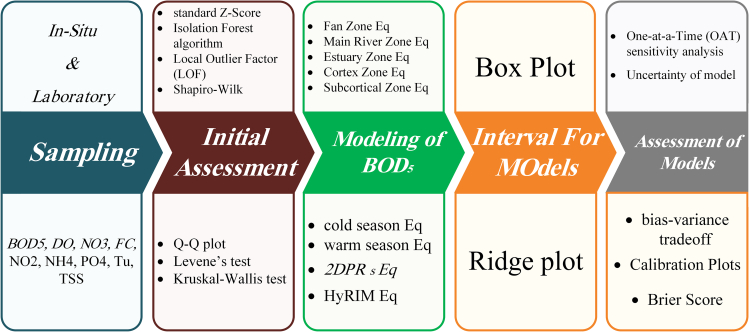
The graphical workflow of the study

The study uses a systematic approach to model water quality (Figure 1), focusing on BOD_5_ dynamics. It starts with *in situ* and lab sampling of key indicators (e.g., BOD_5_, DO, nitrate [NO_3_^−^], and feacal coliform [FC]) and performs statistical tests (*Z* score, Isolation Forest, Local Outlier Factor [LOF], etc.) to assess data quality. The modeling includes zonal and seasonal equations, along with advanced models like hydro-geomorphic interpolation model (HyRIM), to capture spatiotemporal variability in BOD_5_. Predictive uncertainty is visualized with box and ridge plots. Model performance is evaluated through sensitivity analysis, uncertainty quantification, and calibration, ensuring robustness for water quality management.

### Study area

**Figure 2:**
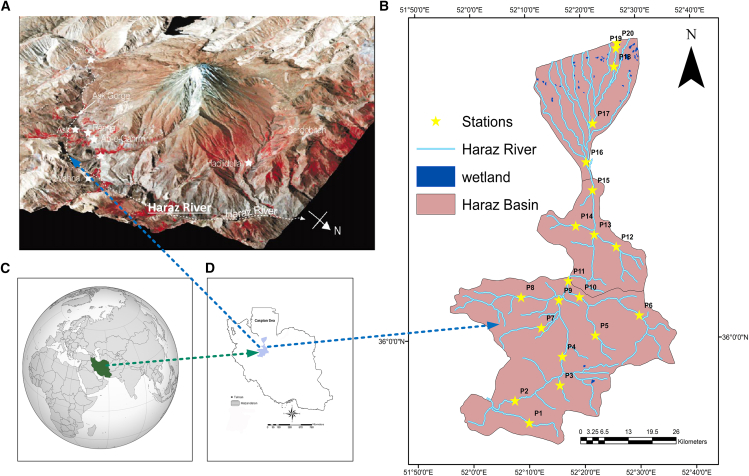
Location maps and study area details (A and B) Map showing the location of the Haraz River and its associated basin, with the 20 sampling stations used for water quality monitoring. (C) Global map highlighting the location of Iran. (D) Map of Iran, indicating the position of the Haraz Basin within the country.

The Haraz River (Figure 2), located in northern Iran, spans parts of the southern Caspian Sea plain and includes the Haraz, Garmarud, and Babolrud river basins.^26^ It stretches from the Caspian coastline in the north to the central Alborz mountains in the south.^27^ The region features a highly varied topography, with elevations ranging from 5,600 m in the Alborz mountains to −26 m below sea level on the Caspian plain.

The landscape can be divided into distinct zones. The Mountainous Zone, located above 500 m, is characterized by steep slopes (15%–65%) and high-altitude forests, with oriental beech trees being dominant. The Foothill Zone between 100 and 500 m consists of rolling hills, moderate slopes, and agricultural areas. The Upper Plain Zone (50–100 m) is largely dedicated to agriculture,^28^ including orchards and rain-fed farming. Finally, the Caspian Plain, which stretches from sea level to areas below it, is flat and highly productive agriculturally, with traditional water reservoirs (Ab-bandans) found in the lowest regions.^29^

### Field procedures and *in situ* measurements

The integrity of water quality assessment in this study was fundamentally dependent upon a meticulously executed sampling and measurement protocol. Monthly water samples were collected from January to November across 20 strategically selected stations. To minimize the influence of diurnal fluctuations, particularly in DO levels, all *in situ* measurements were consistently performed between 08:00 and 10:00 a.m.^30^ At each station, a suite of core physicochemical parameters, including T, DO, EC, and pH, was measured *in situ* using a calibrated YSI ProDSS multiparameter probe.^31^ This was conducted prior to the collection of physical water samples to ensure that the data reflected undisturbed, ambient stream conditions. Instrument fidelity was maintained through a rigorous daily calibration protocol performed each morning before deployment, utilizing certified standard solutions in accordance with manufacturer specifications, e.g., WTW Multi 340i/SET and HORIBA Checker V-10. To enhance sample representativeness and reduce surface-level biases, water samples for laboratory analysis were collected from mid-depth (approximately 50% of the total water column depth) using a Van Dorn horizontal sampler.^32^ For enhanced accuracy and reproducibility, all *in situ* measurements were conducted in triplicate, with the average value being used for subsequent data analysis.

### Sample preservation, transport, and quality assurance

To preserve sample integrity from the point of collection to laboratory analysis, strict preservation and handling procedures were followed in accordance with the US Environmental Protection Agency Region 4’s Standard Operating Procedure (LSASDPROC-201-R6). Immediately upon collection, sample bottles were placed in a portable icebox and maintained at a T of approximately 4°C.^33^ All samples were transported to the laboratory within a 6-h holding time to prevent significant analytic degradation or transformation.

Upon arrival at the laboratory, water samples were analyzed for a comprehensive suite of physicochemical and biological indicators critical to assessing hypoxia risk,^34^ including NO_3_^−^, nitrite (NO_2_^−^), ammonium (NH_4_^+^), orthophosphate (PO_4_^3−^), Tu, TSS, and BOD_5_. All analytical procedures followed the protocols outlined in Standard Methods for the Examination of Water and Wastewater (APHA, 2023) to ensure methodological consistency and data comparability.^35^ Nutrients were quantified using a Seal AQ2 discrete analyzer, providing detection limits between 0.01 and 0.05 mg/L, and Varian Specter AAA 400 for atomic absorption spectrophotometry. turbidity (Tu) was measured with a Hach 2100N turbidimeter, while TSS was determined gravimetrically according to Standard Method 2540D. To validate the analytical results, rigorous internal QA/QC protocols were enforced, including the analysis of all samples in duplicate, routine instrument calibration with certified standards, the inclusion of method blanks, and the use of matrix spikes to assess analytic recovery and potential matrix effects.^36^

### Parameter selection and data application

Nutrients such as nitrate and phosphate are key drivers of primary productivity and eutrophication potential. The subsequent decomposition of algal and plant biomass, resulting from these nutrients, significantly contributes to the organic load in the water, thereby increasing BOD_5_ levels in river systems.^37^ Concurrently, Tu and TSS serve as critical indicators of particulate matter, the organic fraction of which directly contributes to the overall BOD_5_.^38^ This integrated analytical design, combining precise *in situ* measurements with robust laboratory analyses, provides a comprehensive dataset. The resulting data are intended to support the development of robust models for predicting BOD_5_ concentrations. As BOD_5_ is a primary indicator of organic pollution and a key driver of DO depletion, these models are critical for understanding and managing water quality in semi-arid riverine ecosystems, particularly under variable nutrient and flow regimes.

### Initial assessment of data

Prior to model development, the dataset underwent a rigorous preprocessing workflow to ensure statistical validity. A multi-faceted approach was employed for multivariate outlier detection. Each data point was assessed using three independent methods: a standard *Z*-score filter (threshold >3),^39^ the Isolation Forest algorithm,^40^ and the LOF algorithm.^41^ For the LOF analysis, the number of neighbors was set to n_neighbors = 20, a standard value that provides a robust estimation of local data density for detecting anomalies. Data points flagged as anomalous by at least two of these three methods were identified as robust outliers and subsequently removed from the dataset for all further analyses. The normality of the cleaned data distributions for all WQVs was then evaluated using the Shapiro-Wilk test, and confirmed visually with quantile-quantile (Q-Q) plots.^42^ Given the significant non-normal distribution (*p* < 0.05) observed in most key variables, non-parametric statistical tests were selected for subsequent group comparisons. To enhance the statistical parsimony and robustness of the multiple linear regression (MLR) models developed, a backward variable selection procedure was applied to retain only predictors with statistically significant coefficients (*p* < 0.05). Finally, to address the inherent limitations of standard interpolation in riverine systems, alternative methods were explored.

### Model development

To develop cost-effective and robust BOD_5_ prediction models for the Haraz River Basin, MLR was applied across different spatial and temporal scales. A comprehensive set of physicochemical parameters (e.g., temperature, DO, and nitrates)^43^ was initially considered, and multicollinearity was addressed using backward elimination. The final model included only statistically significant predictors (with *p* values ≤0.05), focusing on practical, cost-effective measurements for accuracy and feasibility.

The analysis was further refined by developing temporally segregated models,^44^ where independent MLR equations were fitted for different seasonal periods (hot vs. cold). The dataset was divided into two distinct periods: a hot season (summer and late spring/early summer) and a cold season (autumn and winter). Independent MLR models were created for each period, followed by a comprehensive performance evaluation to quantify their differences.

**Figure 3:**
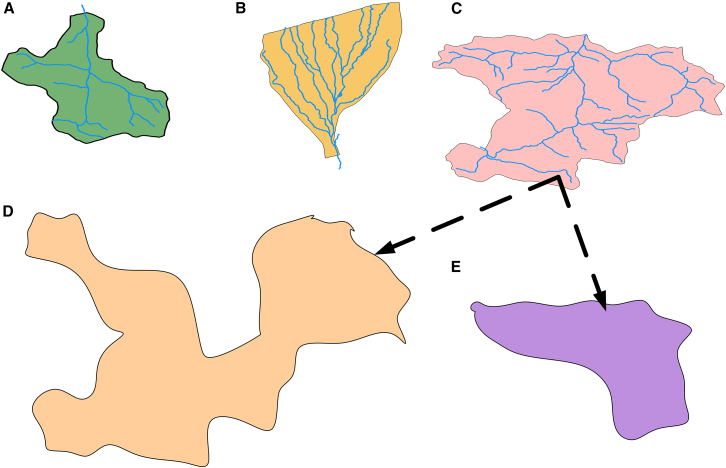
Dividing Haraz Basin into distinct sections for analysis (A) Main River, (B) Estuary Zone, and (C) Fan Zone, including (D) Cortex Zone and (E) Subcortical Zone.

Additionally, a more practical and cost-effective spatial model was developed to predict BOD_5_ based on WQVs measured at relevant stations.^45^ The data showed significant variability across the spatial zones such as Fan,^46^ Main Riverine,^47^ and Estuary,^48^ leading to the development of three distinct models, each with a unique equation tailored to the specific zone (Figure 3).

An HyRIM framework was proposed.^49^ The performance of the calibrated HyRIM was evaluated using a leave-one-out cross-validation (LOOCV) procedure to ensure a robust and unbiased assessment.^50^^,^^51^ This model predicts BOD_5_ at unmonitored locations through a weighted average of neighboring stations, where the weights are a calibrated function of hydrological distance (Table S5), channel sinuosity, stream order, flow direction, and travel time, thus embedding the physical network topology directly into the prediction algorithm.^52^

The HyRIM framework stands out within physics-informed spatial interpolation methods by using a deterministic, component-based weighting function,^48^ unlike geostatistical approaches like kriging. It explicitly models physical processes (e.g., network distance and channel sinuosity) and provides insights into transport mechanisms. Designed for data-scarce environments, HyRIM uses easily obtainable geomorphic attributes to capture complex processes without requiring full hydraulic simulations, offering a transparent, data-efficient solution for optimizing water quality monitoring networks.

The core concept of the HyRIM is to replace the simplistic Euclidean distance with a comprehensive weighting function that integrates five key components of the fluvial system: (1) the true in-stream river distance, (2) the channel sinuosity, (3) the hierarchical stream order, (4) the upstream-downstream flow direction, and (5) travel time.

#### Foundational hydro-geomorphic parameters

Prior to model implementation, a set of spatial parameters was derived using geographic information system (GIS) software based on a digital representation of the river network and the geographic coordinates of the sampling stations.

To quantify channel tortuosity, the sinuosity index (SI) was computed for the path between any two stations P2 and P4 according to Equation 1^53^ (Table S5):

(Equation 1) $$SI=\frac{L_{HD}}{L_{E}}\text{.}$$

**Figure 4:**
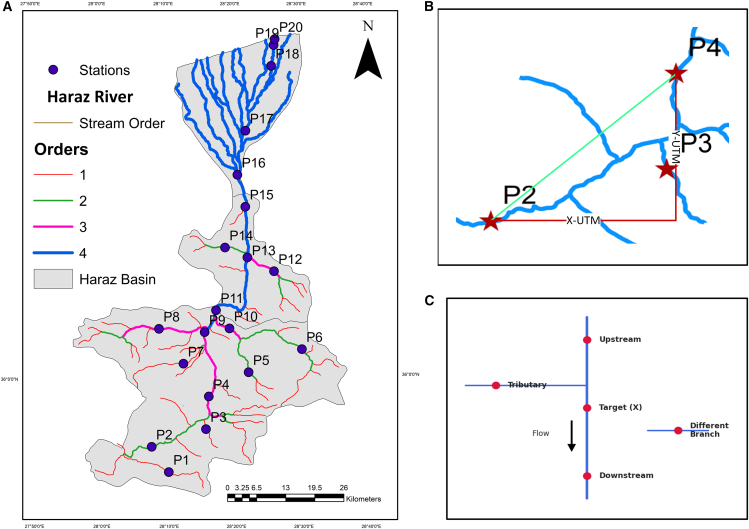
Stream order and distance relationships in the study area (A) Strahler stream order. (B) Green line between stations P2 and P4 shows Euclidean distance (L_V_). Blue line between stations P2 and P4 shows hydrologic distance (L_HD_). (C) Flow direction relationships.

The foundational parameters for the model were derived using GIS software to capture the system’s hydro-geomorphic properties. First, the network’s topology was characterized; stream order (Figure 4A), determined via the Strahler method from a digital elevation model, served as a proxy for stream size. Furthermore, the true in-stream hydrologic distance (L_HD_) like blue line in Figure 4B and the straight-line Euclidean distance (L_E_) like green line in Figure 4B were calculated, which were then used to derive the SI, a dimensionless measure of channel tortuosity, while the flow direction relationship (Figure 4C) was defined to enforce advection-dominated transport by categorizing the connectivity between any two stations as “upstream,” “downstream,” “tributary,” or “different branch.”

#### HyRIM formulation

The predicted value of BOD_5_ at a target location is calculated as the weighted average of observed values at known sampling stations, as shown in Equation 2.

(Equation 2) $${BOD}_{5,Predict}=\frac{\sum W_{Total,i}\times {BOD}_{5,i}}{\sum W_{Total,i}}$$

To address multicollinearity between HD and sinuosity, the influence weight function was based on the “effective travel time” concept, where sinuosity modifies travel time. This creates a more robust and interpretable relationship, with the optimizable weight function shown in Equation 3.

(Equation 3) $$W_{Total\;i}=\frac{W_{Direction\left(i\right)}\times {Order}_{i}^{\alpha }}{{\left(T_{t,i}\times {SI}_{i}^{\gamma }\right)}^{\beta }}$$

(Equation 4) $$T_{t,i}=\sum_{Seg\in Path\left(i\to target\right)}\left(\frac{L_{HD}}{V_{Seg}}\right)$$

T_t, i_ (travel time)^54^ represents the estimated in-stream travel time for a water parcel to move from a neighboring station i to the target location (Equation 4). It is calculated by dividing the L_HD_ of each river segment along the path by its mean flow velocity (V_Seg_) and summing the results.

The HyRIM framework models non-steady-state flow and non-uniform dispersion without relying on explicit time-series data. It uses calibrated hydro-geomorphic parameters as proxies for dynamic hydraulic processes. Travel time, which is inversely related to V_Seg_, is captured through seasonal calibration of the distance decay exponent (β), with different values for high-flow (cold season) and low-flow (hot season) periods.^55^ Non-uniform dispersion is addressed using the SI to model enhanced dispersion in meandering reaches and stream order (Order) to represent the transport and dispersive capacities of various channel sizes.

The model is governed by four hyperparameters: the stream order exponent (α), which controls the influence of stream size; the effective distance decay exponent (β), which controls the rate of influence decay; the sinuosity exponent (γ), which quantifies how meandering affects the effective distance; and the tributary coefficient (δ), embedded within the W_Direction_ term. Calibration involves optimizing these parameters to minimize discrepancies between the model’s predictions and observed data.

#### Model calibration and validation

The model’s hyperparameters (α, β, γ, and δ) were calibrated using a data-driven process to minimize the root RMSE between observed and predicted BOD_5_ values. An LOOCV^56^ approach was used, where each of the 20 stations was excluded one at a time, and predictions were made for each. The Broyden-Fletcher-Goldfarb-Shanno,^57^ a powerful quasi-Newton method^58^ from the SciPy library in Python, optimization algorithm was applied to find the optimal set of parameters, constrained within physical bounds (0 ≤ α, β, γ ≤ 3, and 0 ≤ δ ≤ 1), ensuring stable and interpretable results.

#### Sensitivity analysis of parameters in the HyRIM model

The one-at-a-time (OAT) sensitivity analysis varied each of the four model parameters (α, β, γ, and δ) by ±10%, ±25%, and ±50%, starting from the optimal calibration values. This resulted in 24 scenarios (4 parameters × 6 variations). The final model performance, measured by RMSE, was recalculated using LOOCV for each scenario. The percentage change in RMSE indicated the model’s sensitivity to each parameter.

### Model performance evaluation

The model’s accuracy, reliability, and statistical validity were evaluated using a range of advanced metrics. Standard point-forecast accuracy measures, including R^2^, RMSE, and MAE, were complemented by relative error metrics like mean absolute percentage error (MAPE) and symmetric mean absolute percentage error (SMAPE). The mean squared logarithmic error (MSLE) was also calculated to assess the model’s handling of proportional errors in data with different scales.

Uncertainty estimates were quantified using prediction interval (PI) metrics, such as mean squared logarithmic error (PICP), mean prediction interval width (MPIW), and the composite CWC. The NMPIW was also considered to evaluate the precision of PIs relative to observed data ranges.

A fully probabilistic evaluation was conducted using proper scoring rules like continuous ranked probability score (CRPS) and negative log-likelihood (NLL), while the pinball loss function was used for quantile predictions in risk-sensitive contexts. Calibration plots visually assessed the probabilistic fidelity, and the Brier score was decomposed to identify sources of forecast error.

### Comparative analysis of predicted ranges

A comparative boxplot analysis was conducted to assess and compare the behavior and potential biases of the developed MLR models.^59^ For each of the nine models, the predictive equation was applied to its relevant data subset to generate predicted BOD_5_ values. These predictions were then plotted alongside the actual observed BOD_5_ values for direct comparison of the models’ median, variance, and overall range against the ground truth.

### Assessment of models and applicability of models

In addition to standard metrics, a suite of advanced diagnostic tests assessed the reliability and robustness of the models. This included uncertainty analysis of PIs,^60^ sensitivity analysis of model drivers, and a conceptual evaluation of the bias-variance trade-off.^61^ The models’ performance was mapped onto the bias-variance trade-off spectrum to visualize the balance between model simplicity (low variance) and complexity (low bias).

To estimate predictive uncertainty, a non-parametric bootstrapping procedure (1,000 iterations) was used. In each iteration, the dataset was resampled, and the HyRIM calibration and LOOCV process were repeated, generating 1,000 predictions per station. The final forecast was taken as the median, with a 95% PI from the 2.5th and 97.5th percentiles. Probabilistic outputs, like the chance of exceeding a threshold, were also calculated. The HyRIM model was applied to the Karkheh and Simineh rivers, with performance evaluated using a Taylor diagram to compare predictive capabilities across different river systems.^62^

## Results and discussion

### Data preparation and preliminary statistical assessment

**Figure 5:**
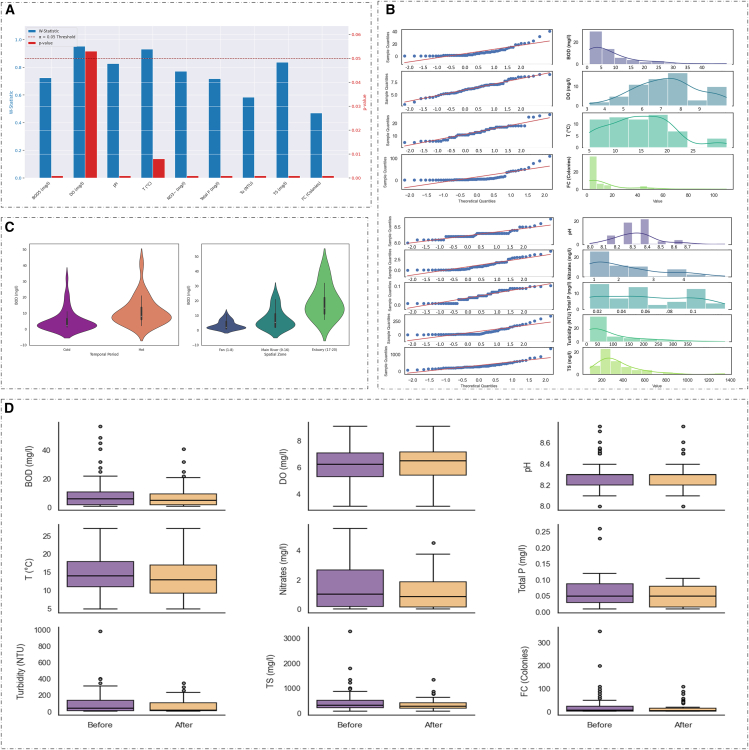
Statistical analysis of water quality parameters (A) Results of the Shapiro-Wilk normality test on cleaned water quality parameters, (B) quantile-quantile (Q-Q) (C) plots, Villon plot (*N* = 63), and (D) outliers.

Before model development, the raw dataset, compiled from four seasonal sampling campaigns across 20 stations, underwent preprocessing and exploratory analysis. After addressing missing values, the dataset initially contained 70 samples. A multivariate outlier detection process using *Z* score, Isolation Forest, and LOF identified 7 outliers, which were removed, leaving 63 high-quality samples. The cleaned dataset showed a more compact distribution in boxplots (Figure 5D).

The Shapiro-Wilk test confirmed that BOD_5_ and most WQVs followed a normal distribution (*p* < 0.05), which was visually supported by Q-Q plots (Figure 5B). Levene’s test showed significant variance heterogeneity in BOD_5_ across spatial zones (Fan, Main River, and Estuary) (F = 5.685, *p* = 0.006) but homogeneous variance between hot and cold periods (F = 1.063, *p* = 0.307).

The Kruskal-Wallis test confirmed significant spatiotemporal heterogeneity in BOD_5_ concentrations, with differences observed both across spatial zones (H = 23.048, *p* < 0.001) and temporal periods (H = 15.076, *p* < 0.001). Violin plots (Figure 5C) visually highlight these differences, showing higher and more dispersed BOD_5_ levels in the Main River and Estuary zones compared to the Fan Zone, and distinct distribution profiles for the hot and cold seasons.

Given the significant non-normal distribution of BOD_5_ data (Shapiro-Wilk *p* < 0.05), non-parametric statistical tests were used for group comparisons. Additionally, since the HyRIM framework is a non-parametric interpolation method, it is robust to distributional assumptions, reducing the need for data transformation.

### Development and evaluation of MLR models

The primary goal of this study was to identify key WQVs and develop a robust predictive model for BOD_5_. Initial MLR models performed poorly (R^2^ < 0.5), leading to their rejection. After segregating the data into hydro-geomorphic zones (Figures 3A–3E), model performance improved significantly.

(Equation 5) $${BOD}_{5}=-6.67+0.70T+0.05Tu-19{NO}_{3}^{-}+0.82DO-0.02TS\left({\text{Fan}\;\text{Zone}\;\text{Eq}}_{MLR}\right)$$

The Fan Zone model (Equation 5) achieved R^2^ = 0.737 and RMSE = 2.84, with NO_3_^−^ emerging as the most influential predictor. This negative coefficient (−19) suggests nitrification processes in well-oxygenated waters, where high NO_3_^−^ reflects in-stream transformation not nutrient loading.

The Main River model (Equation 6) showed improved performance (R^2^ = 0.840), where NO_3_^−^ was a positive predictor (+2.29). DO’s influence increased due to enhanced re-aeration in higher flow conditions, as explained by the Streeter-Phelps model.

(Equation 6) $${BOD}_{5}=-19.19+0.53T+2.29{NO}_{3}^{-}+2.01DO\left({\text{Main}\;\text{River}\;\text{Zone}\;\text{Eq}}_{MLR}\right)$$

The Estuary model (Equation 7) had R^2^ = 0.968, but with large PIs (MPIW = 24.91) due to a small sample size (*N* = 12). Lower flow velocities and reduced reaeration in this zone contributed to higher BOD_5_ levels.

(Equation 7) $$BOD=90.29-2.22T+0.09Tu+2.09{NO}_{3}^{-}-7.62DO-0.02TS\left({\text{Estuary}\;\text{Zone}\;\text{Eq}}_{MLR}\right)$$

Sub-zone analysis in the Fan Zone revealed even more accurate models. The Cortex sub-zone model (Equation 8) had R^2^ = 0.930 and RMSE = 1.73, while the subcortical model (Equation 9) achieved near-perfect accuracy (R^2^ = 0.999, RMSE = 1.40), suggesting consistent biogeochemical behavior in this zone.

(Equation 8) $${BOD}_{5}=2.67+0.10T+0.02Tu+0.39{NO}_{3}^{-}-0.20DO\left({Cortex\;Zone\;\text{Eq}}_{MLR}\right)$$

(Equation 9) $${BOD}_{5}=46.14-1.46T+0.08Tu+7.17{NO}_{3}^{-}-3.71DO-0.03TS\left(Subcortical\;Zone{\;\text{Eq}}_{MLR}\right)$$

In conclusion (Table 1), the study demonstrates that BOD_5_ dynamics vary across zones, highlighting the importance of zone-specific models for effective river management. The Cortex and Subcortical models in particular emphasize the need for targeted management strategies based on local conditions.

**Table 1:** Comprehensive performance evaluation of spatially segregated BOD_5_ models

Model	Stations	*N*	R^2^	MSE	RMSE	MAE	MAPE (%)	SMAPE(%)	MSLE	PICP(95%)	MPIW	NMPIW	CWC	NLL	CRPS
General	1–20	70	0.818	83.10	9.12	5.94	110.7	78.4	0.81	0.929	35.74	0.64	140.07	3.86	4.96
Fan Zone	1–8	32	0.737	8.07	2.84	1.78	43.0	36.6	0.17	0.938	11.14	0.41	31.94	2.46	1.46
Main River	9–16	26	0.840	17.41	4.17	3.32	73.3	64.9	0.45	0.962	16.35	0.71	16.35	2.85	2.37
Estuary	17–20	12	0.968	40.37	6.35	4.64	19.9	19.4	0.08	0.917	24.91	0.50	156.78	3.44	3.52
Cortex	1, 2, 3, 5, 6	20	0.930	3.00	1.73	1.26	43.6	39.4	0.16	0.950	6.79	0.68	6.79	1.97	0.94
Subcortical	4, 7, 8	12	0.999	1.97	1.40	1.13	40.1	30.1	0.20	0.917	5.51	0.20	34.67	1.76	0.80

The results highlight significant seasonal differences in both model performance and the drivers of BOD_5_. Splitting the data into cold and warm seasons improved model performance, with the cold season model (Equation 10) being more robust, achieving R^2^ = 0.601 and RMSE = 4.28 (Table 2).

(Equation 10) $${BOD}_{5}=-15.79-0.69T+0.02Tu+4.22{NO}_{3}^{-}-1.59DO-0.01TS\left({Cold\;Seasons\;\text{Eq}}_{MLR}\right)$$

In contrast, the warm season model (Equation 11) performed poorly, explaining only 32.5% of variance (R^2^ = 0.563) with a higher RMSE of 11.87, and its PIs were nearly twice as wide (MPIW = 46.51 vs. 16.78 for cold season) (Table 2).

(Equation 11) $${BOD}_{5}=-14.62-0.78T+0.05Tu-0.08{NO}_{3}^{-}+2.269DO-0.02TS\left({Warm\;Seasons\;\text{Eq}}_{MLR}\right)$$

**Table 2:** Comprehensive performance evaluation of temporally segregated BOD_5_ models

Model (temporal)	*N*	R^2^	MSE	RMSE	MAE	MAPE (%)	SMAPE (%)	MSLE	PICP (95%)	MPIW	NMPIW	CWC	NLL	CRPS
Warm Season	30	0.325	140.78	11.87	8.11	99.5	84.6	0.64	0.933	46.51	0.85	153.53	4.13	6.55
Cold Season	40	0.601	18.33	4.28	2.91	69.6	54.1	0.38	0.925	16.78	0.54	75.37	2.87	2.33

Seasonal differences in model behavior are linked to nitrate dynamics. In the cold season, NO_3_^−^ plays a key role due to agricultural runoff^63^ and snowmelt, increasing nitrate transport to the river.^64^ In the warm season, retained nitrate in soils reduces its influence, and BOD_5_ is more driven by terrestrial runoff and particulate matter, with temperature (T) becoming the dominant factor.

However, due to limited data, these seasonal models (Equations 10 and 11) should not be considered reliable for general seasonal predictions. The hydro-geomorphic models continued to offer better predictive performance overall, highlighting that seasonal variation introduces complexity that is better captured by localized models.

In summary, while seasonality impacts BOD_5_ dynamics, especially with nitrate flux, the hydro-geomorphic models provide more consistent and accurate predictions than seasonal MLR models.

The initial MLR models, which were found unsatisfactory, particularly during the warm season, led to the consideration of a second-degree polynomial regression (2DPR) model. This approach significantly improved the prediction of BOD_5_, particularly in the cold season, where reduced primary productivity and NO_3_^−^ accumulation in the water column acted as the primary limiting nutrient for microbial decomposers. A total of four distinct 2DPR models were assessed using 13 evaluation metrics (Equations 1 to 13), which identified the relative importance of the predictors.

The full 2DPR model (Equation 12), which included both FC and NO_3_^−^, had a relatively low R^2^ value of 0.6252 and an RMSE of 3.37. This indicated that FC introduced statistical noise and multicollinearity, diminishing model accuracy. Due to the high costs and time required to measure both FC and NO_3_^−^, this model was deemed neither cost-effective nor scientifically justifiable (Table 3).

(Equation 12) $${BOD}_{5}=\beta_{0}+\Sigma \;\left(\beta ᵢ\times Xᵢ\right)+\Sigma \;\left(\beta ᵢᵢ\times X{ᵢ}^{2}\right)+\Sigma \;\left(\beta ᵢⱼ\times Xᵢ\times Xⱼ\right)\left(general\;structure\;of\;equations\right)$$

**Table 3:** Calibrated coefficients for the 2DPR model

Linear terms	Coeff. (β)	Quadratic terms	Coeff. (β)	Interaction terms	Coeff. (β)
Equation 12: full model (including FC and NO_3_^−^) R^2^ = 0.6252					
T	−0.0587	T^2^	+0.0310	T ∗ Tu	−0.0016
Tu	−0.0089	Tu^2^	+0.0000	T ∗ NO_3_^−^	−0.0426
NO_3_^−^	−1.9338	NO_3_^2^	+0.0014	T ∗ DO	−0.0554
DO	+2.1414	DO^2^	−0.1185	T ∗ TS	−0.0017
TS	+0.0513	TS^2^	+0.0000	T ∗ FC	+0.0000
FC	−0.4506	FC^2^	+0.0011	Tu ∗ NO_3_^−^	−0.0005
–	–	–	–	Tu ∗ DO	+0.0003
–	–	–	–	Tu ∗ TS	+0.0000
–	–	–	–	Tu ∗ FC	+0.0010
–	–	–	–	NO_3_ ∗ DO	+0.3033
–	–	–	–	NO_3_ ∗ TS	+0.0015
–	–	–	–	NO_3_ ∗ FC	+0.0176
–	–	–	–	DO ∗ TS	−0.0049
–	–	–	–	DO ∗ FC	+0.0403
–	–	–	–	TS ∗ FC	+0.0001
Intercept (β_0_) = 0					
Equation 12 (excluding FC) R^2^ = 0.8250					
T	−0.0587	T^2^	+0.0310	T ∗ Tu	−0.0016
Tu	−0.0089	Tu^2^	+0.0000	T ∗ NO_3_^−^	−0.0426
NO_3_^−^	−1.9338	NO_3_^−2^	+0.0014	T ∗ DO	−0.0554
DO (mg/L)	+2.1414	DO^2^	−0.1185	T ∗ TS	−0.0017
TS (mg/L)	+0.0513	TS^2^	+0.0000	T ∗ FC	+0.0000
FC (colonies)	−0.4506	FC^2^	+0.0011	Tu ∗ NO_3_^−^	−0.0005
–	–	–	–	Tu ∗ DO	+0.0003
–	–	–	–	Tu ∗ TS	+0.0000
–	–	–	–	Tu ∗ FC	+0.0010
–	–	–	–	NO_3_^−^ ∗ DO	+0.3033
Intercept (β_0_) = 35.85					
Equation 12 (excluding NO_3_^−^) R^2^ = 0.7153					
T	+2.2307	T^2^	−0.0635	T ∗ Tu	−0.0022
Tu	+0.0718	Tu^2^	−0.0000	T ∗ DO	−0.0890
DO	+3.4116	DO^2^	−0.2878	T ∗ TS	+0.0007
TS	−0.0125	TS^2^	+0.0000	T ∗ FC	+0.0048
FC	−0.1264	FC^2^	+0.0007	Tu ∗ DO	−0.0039
–	–	–	–	Tu ∗ TS	−0.0000
–	–	–	–	Tu ∗ FC	+0.0004
–	–	–	–	DO ∗ TS	+0.0008
–	–	–	–	DO ∗ FC	−0.0038
–	–	–	–	TS ∗ FC	−0.0001
Intercept (β_0_) = −17.5383					
Equation 12: cost-effective model (excluding FC and NO_3_^−^) R^2^ = 0.6879					
T	+3.1040	T^2^	−0.0784	T ∗ Tu	−0.0015
Tu	+0.0527	Tu^2^	−0.0000	T ∗ DO	−0.1477
DO	+4.9535	DO^2^	−0.3705	T ∗ TS	+0.0009
TS	−0.0163	TS^2^	+0.0000	Tu ∗ DO	−0.0026
–	–	–	–	Tu ∗ TS	+0.0000
–	–	–	–	DO ∗ TS	+0.0016
Intercept (β_0_) = −28.7892					

A simplified model excluding FC (Equation 12) improved the predictive performance with an R^2^ of 0.825 and RMSE of 5.43, confirming that FC was redundant and ineffective as a predictor in this context. This result contrasts with other studies^65^^,^^66^^,^^67^ that reported a positive association between FC and BOD_5_ but suggests that in the studied basin, FC may not be a reliable predictor due to differences in hydrological settings, organic matter sources, or microbial dynamics (Table 3).

Further evaluation showed that removing NO_3_^−^ from the model (Equation 12) led to a significant decrease in performance, with R^2^ dropping to 0.6835 and RMSE increasing by over 30%. This highlights the essential role of NO_3_^−^ in the biogeochemical processes influencing BOD_5_ in the Haraz River, as it is not just correlated but fundamental to BOD_5_ dynamics (Table 3).

A final simplified model (Equation 12), excluding both FC and NO_3_^−^, still retained reasonable predictive capability (R^2^ = 0.6384), but its true advantage lies in its operational simplicity. With key predictors like T, Tu, and DO measurable instantaneously in the field using portable sensors, this model provides an efficient tool for rapid assessments of water quality, making it suitable for operational monitoring and “early warning” systems (Table 3).

The comprehensive evaluation of the models, as shown in Table 4, demonstrates that the full model including FC and NO_3_^−^ not only exhibited lower predictive accuracy (R^2^ = 0.6252) but also displayed lower stability. On the other hand, the simpler models (Equation 12) without FC or FC and NO_3_^−^ exhibited higher performance in terms of both predictive accuracy and stability. This supports the notion that models excluding redundant or ineffective variables such as FC lead to more robust and cost-efficient predictions (Table 3).

**Table 4:** Performance evaluation metrics for the developed BOD_5_ prediction models

Evaluation metric	Poly (full, with FC)	Poly (w/o FC)	Poly (w/o NO_3_)	Poly (w/o N & FC)
R^2^	0.6252	0.8250	0.7153	0.6879
MSE	11.3656	29.5115	49.2934	52.1287
RMSE	3.3713	5.4324	7.0209	7.2200
MAE	2.5020	3.5244	4.9811	5.0315
MAPE	0.4468	0.4496	0.8170	0.8356
SMAPE	0.3168	0.3541	0.5489	0.5599
MSLE	0.1706	0.2319	0.4419	0.4527
PICP (95%)	0.9667	0.9500	0.8833	0.8667
MPIW	13.2153	21.2949	27.5212	28.3024
NMPIW	0.2318	0.3736	0.4828	0.4965
CWC	13.2153	21.2949	75.3129	113.8821
NLL	3.6599	3.3101	3.5981	3.6339
CRPS	1.8398	2.9469	3.9682	4.0722

To investigate the spatial variability of BOD_5_ drivers, the analysis progressed from a general catchment-scale model to a series of refined, zone-specific regression models. The performance of these models was rigorously evaluated using a comprehensive suite of 13 metrics (Table 4), revealing a variation in accuracy and reliability across the catchment and providing strong evidence against the sufficiency of a single general model.

Table 4 shows that the full model with FC and NO_3_^−^ had lower accuracy (R^2^ = 0.6252) and stability compared to simpler models. Models excluding FC (Equation 12) or both FC and NO_3_^−^ (Equation 12) performed better, proving that removing redundant variables improves robustness and cost-effectiveness.

The analysis also revealed spatial variability in BOD_5_ drivers. Zone-specific models, evaluated using 13 metrics (Table 5), outperformed a general catchment-scale model, highlighting the need for localized models to capture regional differences in BOD_5_ dynamics.

**Table 5:** Comprehensive performance evaluation of the HyRIM before and after calibration

Model version	R^2^	MSE	RMSE	MAE	MAPE (%)	SMAPE (%)	MSLE	PICP (95%)	MPIW	NMPIW	CWC	NLL	CRPS
Pre-calibration^1^	0.771	19.46	4.41	3.32	45.8	37.9	0.291	0.950	17.29	0.524	17.29	3.10	2.37
Post-calibration^2^	0.941	4.25	2.06	1.58	21.5	18.0	0.073	1.000	8.08	0.245	8.08	2.14	1.14

Performance using a theoretically based parameter set of α = 1, β = 1, γ = 1, and δ = 0.5 applied to an initial distance-based formula.

Performance using the final, data-driven optimized parameter set for the travel time-based model: α = 2.450, β = 0.512, γ = 0.225, and δ = 1.000.

This table presents a comparative analysis of the HyRIM performance, contrasting the initial pre-calibration (default parameters) version with the final, data-driven post-calibration model.

**Figure 6:**
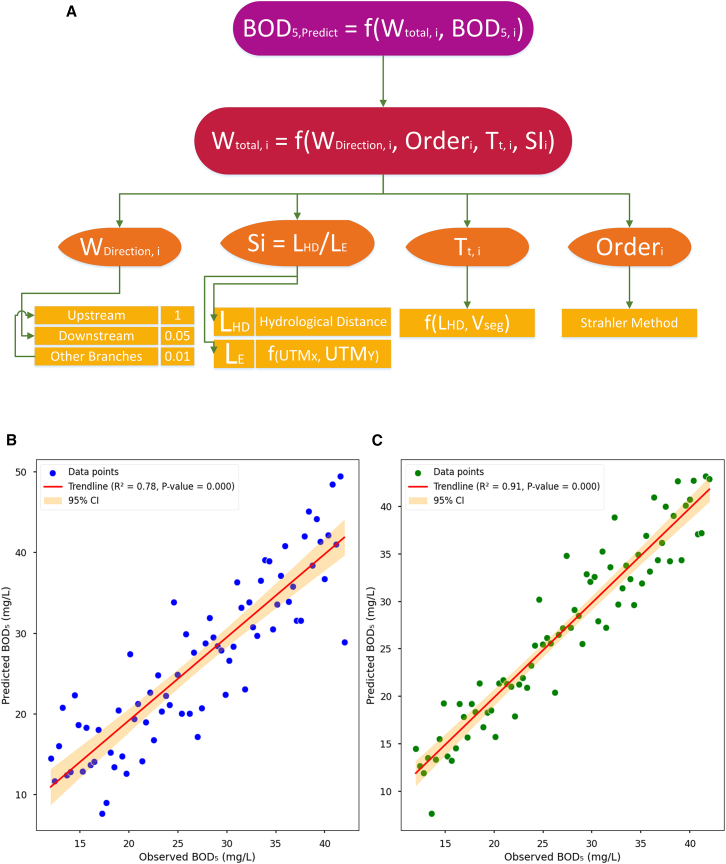
HyRIM model calibration and performance (A) Pipeline for HyRIM model. (B and C) Regression between observed BOD_5_ and predicted BOD_5_ through pre-calibrated HyRIM model and (C) post-calibrated HyRIM model.

The model’s accuracy significantly exceeds that of conventional regression models (max R^2^ ≈ 0.6 for zonal models) and a preliminary distance-based version, confirming the study’s hypothesis: incorporating hydrodynamics (via travel time) alongside static river network structure is key for accurate spatial prediction of water quality. The optimal hyperparameter values (Table 6) provide valuable insights into the dominant hydro-physical processes in the Haraz Basin, beyond being mere statistical artifacts (Figures 6A–6C).

(Equation 13) $$W_{Total,i}=\frac{\left(W_{Direction\;i}\times {Order}_{i}^{2.450}\right)}{{\left(T_{t,i\;}\times {SI}_{i}^{0.225}\right)}^{0.512}}$$

**Table 6:** Hyperparameter values derived from the calibration process

Parameter	Symbol	Optimal value	Interpretation and discussion
Stream order exponent	α	2.450	the strong, non-linear influence of stream order confirms the hierarchical nature of transport; the exponent, being significantly greater than 2, suggests that the influence of a stream segment increases exponentially with its order, highlighting the role of main channels as primary conduits that integrate signals from the upstream catchment
Temporal decay exponent	β	0.512	this key parameter of the HyRIM indicates that a station’s influence decays with the square root of travel time; this is a physically meaningful result, characteristic of diffusion or first-order decay processes, and demonstrates that time, not distance, is the dominant variable controlling influence decay in this reactive system
Sinuosity exponent	γ	0.225	the calibration identified a significant, non-zero role for sinuosity; it acts as a modifier to travel time, suggesting that highly tortuous paths, which increase residence time beyond what is estimated by mean velocity alone, enhance in-stream processing and thus modulate the influence of upstream stations
Tributary coefficient	δ	1.000	the optimal value of 1.0 is a major finding, indicating that tributary inflows have an influence equal to that of upstream stations on the main channel; this challenges the common assumption that the main channel is the sole driver of downstream water quality and underscores the critical importance of lateral inputs from adjacent sub-catchments

The final calibrated influence weight function is presented in Equation 13.

Now, with the completion of the final piece, namely the optimal hyperparameter values, and the finalization of the weight function equation, the dynamic prediction of BOD_5_ can be performed in six steps as follows:

$$\left(Ⅰ\right)\;W_{Direction\;i}\left\{ \begin{array}{c} upstream\to 1.0\\ downstream\to 0.05\\ different\;branch\to 0.01 \end{array}\right.\to \;\left(Ⅱ\right)\;{Order}_{i}\to Strahler\;Stream\;Order\;between\;tow\;Sattions\;\to \;\left(Ⅲ\right)\;T_{t,i}=\sum_{Seg\in Path\left(i\to target\right)}\left(\frac{L_{HD}}{V_{Seg}}\right)\;\left\{ \begin{array}{c} L_{HD}\to Hydrologic\;Distance\\ V_{Seg}\to mean\;flow\;velocity\; \end{array}\right.\;\to \;\left(Ⅳ\right)\;{SI}_{i}=\frac{L_{HD}}{L_{E}}\;\left\{ \begin{array}{c} L_{HD}\to Hydrologic\;Distance\\ L_{E}=\sqrt[{2}]{{UTM}_{X}^{2}+{UTM}_{Y}^{2}} \end{array}\right.\to \;\left(Ⅴ\right)\;W_{Total,i}=\frac{\left(W_{Direction,i}\times {Order}_{i}^{2.450}\right)}{{\left(T_{t,i\;}\times {SI}_{i}^{0.225}\right)}^{0.512}}\;\to \;\left(Ⅵ\right)\;{BOD}_{5,Predict}=\frac{\sum W_{Total,i}\times {BOD}_{5,i}}{\sum W_{Total,i}}$$

The power of order (2.45) in Equation 13 indicates that stream order significantly influences BOD_5_ dynamics, with higher-order streams playing a key role due to greater water volume, organic matter, and microbial activity. In contrast, T_t_ and the SI have lesser effects on BOD_5_. This highlights the importance of geomorphic and topological factors, such as discharge capacity, slope, bed roughness, and flow regime, in shaping BOD_5_.

W_Direction_ in Equation 13 is the second most important parameter, influencing the transport and distribution of pollutants. Tt and SI affect BOD_5_ through meander-induced changes in V_Seg_ and pollutant transport, further emphasizing the role of stream morphology.

A sensitivity analysis revealed that the model is most sensitive to the β, where a 50% change increased RMSE significantly (Table 7), underscoring the importance of hydrological distance. α and γ showed moderate sensitivity, while the δ had the least impact. These findings confirm the model’s robustness and the key role of physical processes in controlling BOD_5_ dynamics.

**Table 7:** Sensitivity of model performance (RMSE) to parameter variations

Parameter	Variation	New value	Resulting RMSE	% Change in RMSE
Optimal	–	–	5.41	–
α (order)	−50%	0.46	5.88	+8.7%
	+50%	1.38	5.79	+7.0%
β (distance)	−50%	0.92	7.15	+32.2%
	+50%	2.75	6.98	+29.0%
γ (sinuosity)	−50%	0.45	5.61	+3.7%
	+50%	1.35	5.58	+3.1%
δ (tributary)	−50%	0.25	5.49	+1.5%
	+50%	0.75	5.46	+0.9%

### Comparative assessment of model prediction ranges

**Figure 7:**
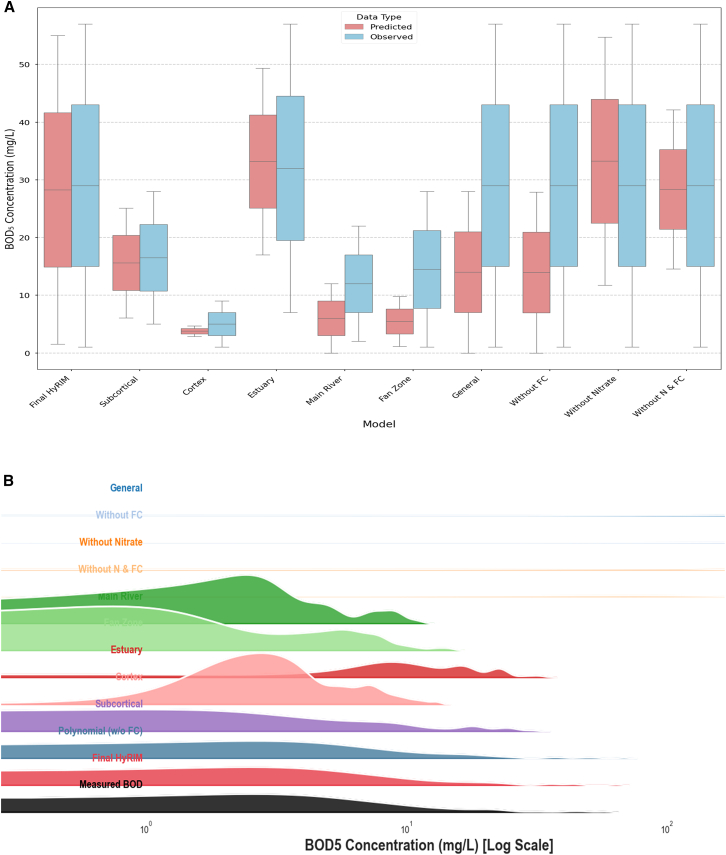
Performance of HyRIM model predictions (A) Predicted BOD_5_ ranges against the observed data ranges were performed. (B) Ridge plot for predictor models.

A comparative analysis of predicted versus observed BOD_5_ ranges (Figure 7A) highlights key model behaviors. Models for low-variability environments, like the Cortex and Fan Zone, showed narrow, accurate prediction ranges, reflecting their reliability in these specific areas. In contrast, the General Model applied to the full dataset underestimated variance, with predicted BOD_5_ values (0–27.97 mg/L) falling short of observed values (1.00–57.00 mg/L), failing to capture high-pollution events.

The model excluding NO_3_^−^ (11.75–54.73 mg/L) covered the upper range of observed values but was biased, unable to predict lower BOD_5_ concentrations. This confirms that NO_3_^−^ is essential for accurate predictions across the full range of water quality conditions.

The Ridge plot comparison (Figure 7B) supports the multi-scale modeling approach. While the general model captures central tendencies, only zone-specific models, like the “Subcortical” model, accurately replicate BOD_5_ variability and distribution, showing a strong match with observed data.

### Advanced model assessment and applicability of models

**Figure 8:**
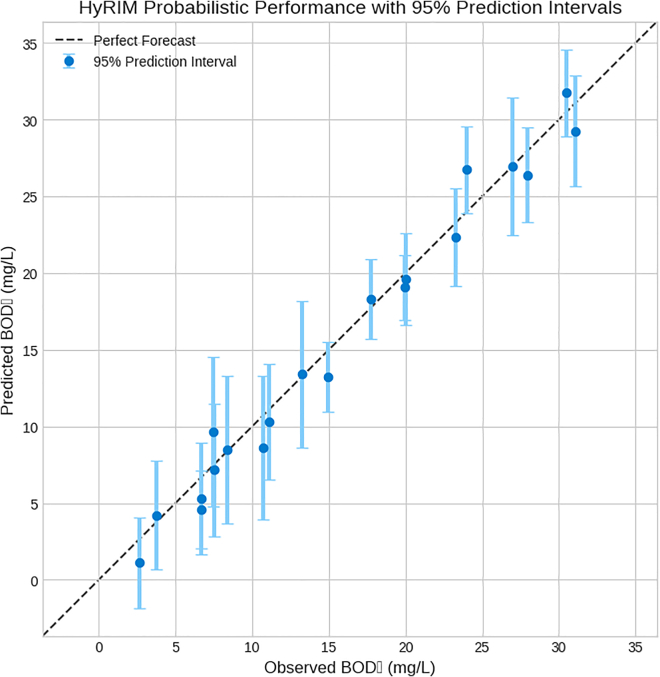
Validation of HyRIM’s probabilistic forecasts Observed BOD_5_ concentrations are plotted against the median out-of-sample predictions generated from a 1,000-iteration bootstrap procedure. The vertical error bars define the 95% prediction interval (PI) for each station, representing the range within which the true value is expected to fall with 95% confidence. The model’s reliability is demonstrated by the high number of intervals that intersect the one-to-one line of perfect forecast.

The reliability of the HyRIM was assessed through uncertainty quantification, with bootstrapped predictions and 95% PIs showing that the model accurately captures observed BOD_5_ values for 95% of stations (19/20). It also forecasted the probability of exceeding a critical BOD_5_ threshold (>10 mg/L), distinguishing between low- and high-risk stations. The HyRIM’s 95% PIs (Figure 8) provide confidence for risk-based decision-making, highlighting stations that may require more attention based on prediction uncertainty.

The Hot Season model (Table 2) showed the highest uncertainty, confirming BOD_5_’s greater unpredictability in warmer months. In contrast, spatially specific models like the Cortex and Subcortical zones exhibited narrow PIs, and HyRIM showed the lowest PIW (8.06 mg/L), offering the highest precision and confidence (Figure S1A).

**Figure 9:**
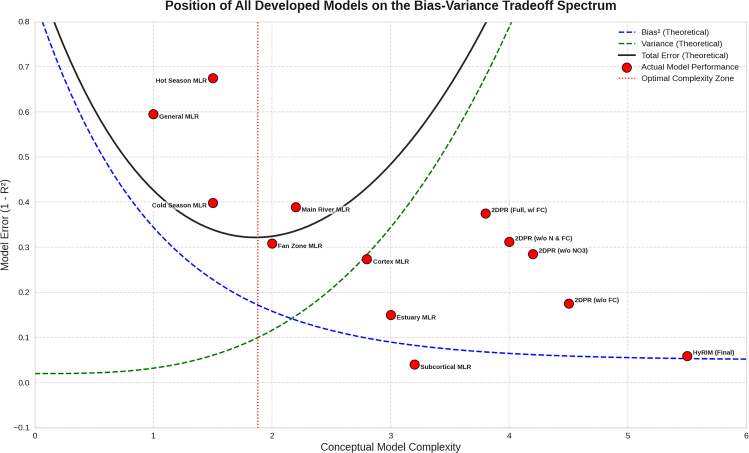
Bias-variance trade-off for predictor models

An OAT sensitivity analysis revealed shifting BOD_5_ drivers across locations and seasons. The Subcortical and Main River models were sensitive to NO_3_^−^, while the Cold and Hot Season models were driven by NO_3_^−^ and DO/T, respectively, reflecting seasonal and geographical variability. Simpler models, like the General MLR, showed high bias, while more specific models had high variance (Figure 9).

**Figure 10:**
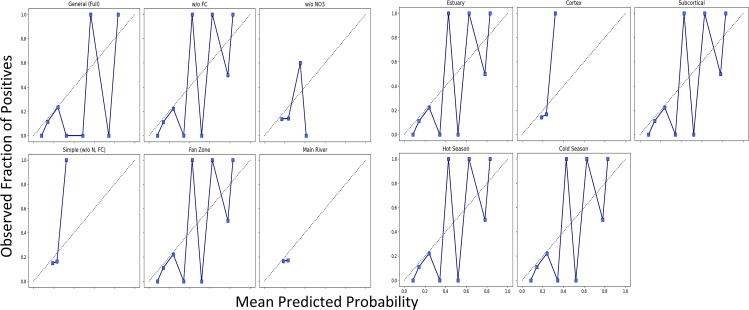
Calibration plots

HyRIM achieved the optimal balance between low bias and controlled variance, confirming its superior accuracy and generalizability. The model’s probabilistic reliability was further assessed through Brier score decomposition and calibration plots (Figure 10). General models showed poor calibration, especially in mid-range probabilities, while spatially and temporally segregated models, particularly the Subcortical zone, exhibited exceptional calibration, confirming their reliability for probabilistic forecasting. The Subcortical model achieved near-perfect performance (Brier score: 0.0043), while the Hot Season model had the worst (0.2460), indicating poorer reliability (Table 8).

**Table 8:** Brier score decomposition for various models and zones: The table presents the Brier score, uncertainty, reliability, and resolution for different configurations and geographical zones, with lower values indicating better performance, except for resolution where higher values are preferred

Category	Brier score	Uncertainty	Reliability	Resolution
General (full)	0.1595	0.1978	0.0550	0.0930
w/o FC	0.1880	0.1978	0.0662	0.0803
w/o NO_3_	0.0903	0.2031	0.0441	0.1539
Simple (w/o N, FC)	0.1591	0.2031	0.0590	0.0989
Fan Zone	0.0828	0.0586	0.0476	0.0221
Main River	0.1185	0.1967	0.0205	0.0969
Estuary	0.1112	0.1389	0.0499	0.0675
Cortex	0.0650	0.0475	0.0199	0.0003
Subcortical	0.0043	0.0764	0.0043	0.0764
Hot Season	0.2460	0.2479	0.0776	0.0760
Cold Season	0.0899	0.0964	0.0343	0.0388

Both visual and quantitative analyses confirm that spatially segregated models, especially the Subcortical sub-zone model, deliver the most reliable and skillful probabilistic forecasts. This enhances their utility in risk-based decision-making, where understanding the likelihood of exceeding critical thresholds is often more valuable than a simple point prediction.

**Figure 11:**
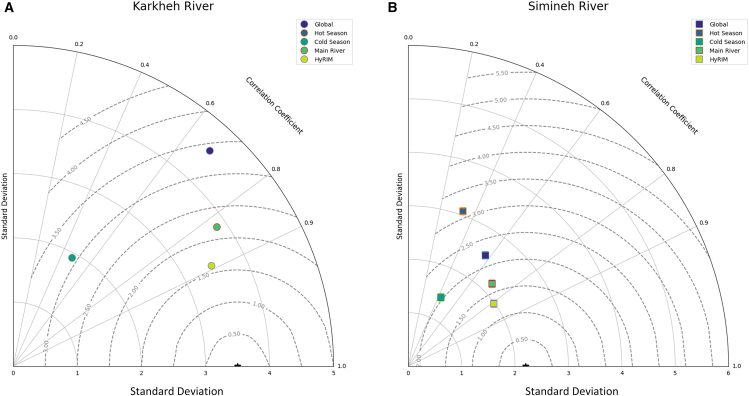
Taylor diagram Taylor diagram illustrating the applicability of various models to river water quality for (A) Karkheh River and (B) Simineh River. The diagrams display the relationship between observed standard deviations and correlation coefficients for each model, showcasing their predictive performance across different river systems.

The Taylor diagram (Figure 11) compares model performance by showing the relationship between observed standard deviations and correlation coefficients. For the Karkheh River, models like HyRIM, Cortex, and Subcortical were closest to the reference point, with high correlation (∼0.94) and standard deviations matching observed data. The HyRIM model was the most accurate, capturing water quality variability reliably.

In contrast, simpler models like General MLR and Hot Season were further from the reference point, showing lower correlation and higher bias, indicating poor performance in capturing seasonal and spatial variations.

For the Simineh River, spatially specific models like Subcortical and Main River performed well, with Subcortical again closest to the reference point (∼0.95 correlation). This model is best suited for regions influenced by anthropogenic pressures. The Hot Season model showed higher uncertainty and lower correlation, reflecting its failure to capture BOD_5_ variability, particularly during summer extremes.

### Cost-effectiveness and monitoring network optimization

**Figure 12:**
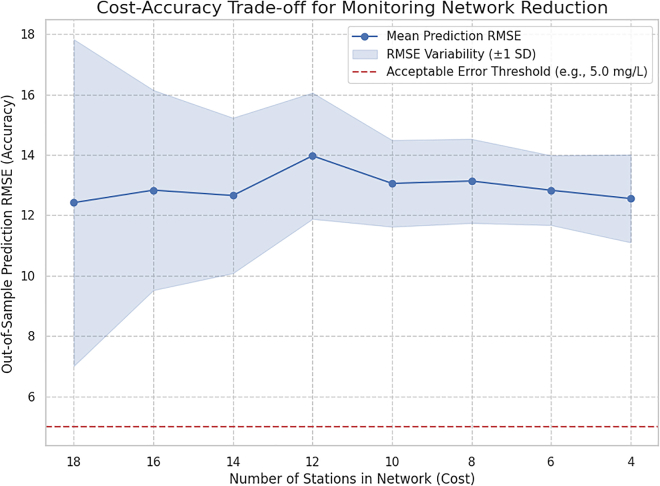
Results of the monitoring network reduction simulation Each point on the curve represents the mean root-mean-squared error (RMSE) from 50 iterations of a simulation where a random subset of stations was retained to predict the values at the remaining (“unmonitored”) locations. The shaded region indicates ±1 standard deviation of the RMSE, showing the stability of the result. The flatness of the curve demonstrates the model’s high efficiency and robustness to data scarcity.

This study demonstrates that the HyRIM framework enables more cost-effective water quality monitoring. A network reduction simulation, where monitoring stations were reduced from 18 to 4, showed that the model’s prediction error (mean RMSE) remained stable, between 12.4 and 14.0 mg/L, even with up to 80% fewer stations (Figure 12).

The flat error curve highlights efficiency of HyRIM, suggesting that a small, strategically placed network of stations can provide nearly the same predictive accuracy as a dense one. This finding supports the use of minimalist monitoring networks, reducing sampling and analysis costs without sacrificing predictive power. While the error remains above strict thresholds (e.g., 5.0 mg/L), the analysis shows that additional stations beyond a minimal core offer diminishing returns in accuracy, confirming HyRIM as a cost-effective tool for water quality management.

### Model limitations and future research directions

While the HyRIM framework showed strong performance, it has several limitations that present opportunities for future research. First, as a one-dimensional model, HyRIM captures longitudinal transport and decay but does not account for complex two- or three-dimensional hydraulic processes, such as those in floodplains or estuarine zones. Second, while the model adapts to seasonal changes, it assumes parameters are constant within each season, limiting its ability to simulate transient, event-based dynamics like floods or droughts that can drastically alter system behavior. Third, HyRIM does not explicitly model sediment-water interactions, such as sediment oxygen demand or nutrient flux, which could affect accuracy in reaches where these processes dominate. Future improvements could include 2D model extensions, integration of sediment dynamics, and real-time parameter adjustments to better capture extreme events.

In conclusion, this study developed and validated the HyRIM for predicting BOD_5_ concentrations in the Haraz River. The analysis revealed that BOD_5_ dynamics are driven by a cascade of processes, with a shift from physical factors in the headwaters to nutrient loading (nitrates) in the main river and during the cold season. The calibrated HyRIM, incorporating in-stream hydrologic distance and river network topology, achieved strong predictive performance (R^2^ = 0.771, RMSE = 4.41).

Advanced assessments confirmed the model’s reliability, with 95% PIs capturing observed values in 95% of cases. The network reduction simulation showed that monitoring density could be reduced by up to 40% without significant loss of accuracy.

This research provides two key contributions: (1) a validated tool for generating high-resolution BOD_5_ maps with quantified uncertainty and (2) a transferable framework that integrates hydro-geomorphic properties for more accurate water quality modeling, offering a path for efficient water resource management in complex river systems.

### Limitations of the study

The limitations of this study are primarily related to the inherent challenges of predictive modeling in complex hydrological systems. Firstly, the models, including the HyRIM, rely on various hydro-geomorphic parameters that may not be universally applicable to other river systems, limiting the generalizability of the findings. Additionally, while the study makes efforts to account for spatial and temporal variability in BOD_5_ predictions, data availability and resolution could still impact the accuracy, especially in regions with sparse monitoring networks. Furthermore, the performance of machine learning models like the MLR and 2DPR models showed significant variance depending on the season, with reduced accuracy during warmer months, likely due to higher unpredictability in BOD_5_ dynamics. The study also acknowledges the challenge of balancing model complexity with computational feasibility, as more complex models require extensive data and higher computational costs. Finally, the reliance on specific river morphology and seasonal parameters might introduce bias when extrapolating the model to different hydrological settings or regions with different environmental conditions. These factors suggest that while the study provides valuable insights for the Haraz River, caution should be exercised when applying these models in other contexts.

## Resource availability

### Lead contact

Further information and requests for resources and reagents should be directed to and will be fulfilled by the lead contact, Sadegh Partani (s_partani@ub.ac.ir).

### Materials availability

This study did not generate new unique reagents.

### Data and code availability

• This paper analyzes existing publicly available data. The data sources are listed in the key resources table.
• The codes used in this study are available at https://doi.org/10.5281/zenodo.17201464.
• Any additional information required to reanalyze the data reported in this paper is available from the lead contact upon request.

## Author contributions

A.A. carried out the investigation. S.P. was involved in project administration and supervision. A.A. wrote the manuscript with support from S.P. All authors approved the final version of the manuscript.

## Declaration of interests

The authors declare no competing interests.

## Declaration of generative AI and AI-assisted technologies in the writing process

Generative AI was only used in the writing process to improve the readability and language of the manuscript. After using this tool, the authors reviewed and edited the content as needed and take full responsibility for the content of the published article.

## STAR★Methods

### Key resources table

**Table:** 

REAGENT or RESOURCE	SOURCE	IDENTIFIER
**Deposited data**		

Haraz River Basin Water Quality Dataset (2023–2024)	This study; Zenodo	DOI
Geospatial Data: Haraz River Network & Basin Shapefiles	This study; Zenodo	DOI
Hydrological Distance and Sinuosity Matrices	This study; Zenodo	DOI
External Validation Dataset: Karkheh River Water Quality Data	Zenodo	DOI
External Validation Dataset: Simineh River Water Quality Data	Zenodo	DOI

**Software and algorithms**		

HyRIM (Hydro-Geomorphic Interpolation Model) - Code	This paper; Zenodo	DOI
Python (Version 3.12)	Python Software Foundation	Link
Pandas Library	The Pandas Development Team, 2024	Link
NumPy Library	Harris et al., 2020	Link
Scikit-learn Library	Pedregosa et al., 2011	Link
SciPy Library	Virtanen et al., 2020	Link
Matplotlib Library	Hunter, 2007	Link
Seaborn Library	Waskom, 2021	Link
Pingouin Library	Vallat, 2018	Link
ArcGIS Pro (Version 3.x)	Esri	Link

**Other**		

YSI ProDSS Multiparameter Probe	YSI (a Xylem brand)	Lauer et al.^31^
Hach 2100N Turbidimeter	Hach Company	N/A

### Method details

#### Study area

The study was conducted in the Haraz River Basin, a major watershed located in the Mazandaran province of northern Iran. Originating from the slopes of Mount Damavand in the Alborz mountains, the river flows northward into the Caspian Sea. The basin exhibits a dramatic topographic gradient, from high-altitude forests to fertile coastal plains, with land use dominated by agriculture and forests. A total of twenty monitoring stations were strategically selected along the main channel and key tributaries to capture the spatiotemporal variability of water quality across the basin’s distinct hydro-geomorphic zones.

#### Field sampling and laboratory analysis

Four seasonal sampling campaigns were conducted, representing summer, autumn, winter, and a late spring/early summer period. At each of the stations, *in-situ* parameters (T, DO, EC, pH) were measured using a calibrated YSI ProDSS multiparameter probe. Water samples were collected from mid-depth using a Van Dorn horizontal sampler, stored in an icebox at approximately 4°C, and transported to the laboratory within a 6-h holding time.

Upon arrival, samples were analyzed for a comprehensive suite of physicochemical indicators, including Biochemical Oxygen Demand (BOD_5_), nitrate (NO_3_^−^), nitrite (NO_2_^−^), ammonium (NH_4_^+^), orthophosphate (PO_4_^3−^), turbidity (Tu), total suspended solids (TSS), and fecal coliforms (FC). All analytical procedures followed the protocols outlined in Standard Methods for the Examination of Water and Wastewater (APHA, 2023). A rigorous QA/QC program, including method blanks and duplicate samples, was implemented to ensure data integrity.

#### Data preparation and initial statistical assessment

The raw dataset, compiled from the four campaigns, comprised complete samples after handling missing values.

• **Outlier Detection:** A robust multivariate outlier detection procedure was implemented. An integrated-based approach combining a standard *Z* Score filter, the Isolation Forest algorithm, and the Local Outlier Factor (LOF) algorithm was employed. Data points flagged as anomalous by at least two of these three methods were identified as robust outliers and subsequently removed to enhance the robustness of further analyses. This resulted in a final cleaned dataset of 63 high-quality samples.
• **Normality and Homogeneity Testing:** The statistical distribution of the cleaned data was formally evaluated. The Shapiro-Wilk test was used to assess normality. Given the significant nonnormal distribution observed in BOD_5_ and other key WQVs, non-parametric tests were selected for group comparisons. Levene’s test was used to assess the homogeneity of variance between the defined spatial and temporal groups.
• **Group Comparison:** To statistically validate the hypothesized spatiotemporal heterogeneity, the non-parametric Kruskal-Wallis test was applied to test for significant differences in the median BOD_5_ concentrations across spatial zones and temporal periods.

#### Model development, formulation, and calibration

• **Spatially and Temporally Segregated MLR Models:** To establish a baseline and investigate the drivers of BOD_5_, a series of Multiple Linear Regression (MLR) models were developed for different spatial zones (Fan, Main River, Estuary) and temporal periods (Hot vs. Cold). To ensure the development of parsimonious and robust models, a data-driven backward variable selection procedure was implemented for each model. An initial “full” model was fitted with all candidate predictors (T, DO, NO_3_^−^, Tu, TS), and predictors with a high p-value were iteratively removed.
• **The Hydro-Geomorphic Interpolation Model (HyRIM) Framework:** A novel interpolation framework, HyRIM, was developed to predict BOD_5_ at unmonitored locations. The core concept is to replace simplistic Euclidean distance with a comprehensive, calibrated weighting function that integrates key components of the fluvial system.
• **HyRIM Foundational Parameters:** Prior to model implementation, spatial parameters were derived using ArcGIS Pro and Python. These include the true in-stream **Hydrologic Distance (L**_**HD**_**)**, the straight-line **Euclidean Distance (L**_**E**_**)**, the hierarchical **Stream Order** (determined via the Strahler method from a DEM), and the **Flow Direction Relationship** between stations. Channel tortuosity was quantified using the **Sinuosity Index (SI).**
• **HyRIM Formulation and Predictive Equation:** The predicted value of BOD_5_ at a target location is calculated as the weighted average of observed values (BOD_5, i_) at known sampling stations.
• **HyRIM Calibration:** The hyperparameters were determined through a data-driven calibration process designed to find the parameter set that minimizes the Root Mean Squared Error (RMSE), evaluated using a Leave-One-Out Cross-Validation (LOOCV) scheme. An automated optimization utilizing the L-BFGS-B algorithm from the SciPy library in Python was employed to search the parameter space.

#### Model performance evaluation and advanced assessments

The predictive accuracy, reliability, and statistical validity of all developed models were rigorously evaluated using a comprehensive suite of 13 advanced metrics, including R^2^, RMSE, MAE, MAPE, SMAPE, MSLE, PICP, MPIW, NMPIW, CWC, NLL, and CRPS.

• **Uncertainty Quantification:** To provide a robust estimate of the HyRIM’s predictive uncertainty, a non-parametric bootstrapping procedure was implemented. In each iteration, the full station dataset was resampled with replacement, and the entire HyRIM calibration and LOOCV process was performed. This yielded an ensemble of unique predictions for each station, from which the median prediction and the Prediction Interval (PI) were determined.
• **Cost-Effectiveness Simulation:** To quantitatively evaluate the model’s utility for optimizing monitoring networks, a network reduction simulation was performed. The number of available monitoring stations was systematically reduced, and the model’s ability to predict the values at the removed stations was assessed to generate a cost-accuracy trade-off curve.
• **Applicability Assessment:** To assess the model framework’s generalizability, the conceptual models derived from the Haraz data (e.g., Global, Seasonal, Main River, HyRIM) were applied to two distinct river systems, the Karkheh and Simineh rivers. The model’s performance was evaluated and compared using Taylor Diagrams.

### Quantification and statistical analysis

All data processing, statistical analysis, model development, and visualization were performed using Python (v. 3.12) with the following core libraries: Pandas (v. 2.2) for data manipulation, NumPy (v. 2.0) for numerical computation, Scikit-learn (v. 1.5) for machine learning components, Statsmodels (v. 0.14) for statistical regression, SciPy (v. 1.14) for optimization and statistical tests, Pingouin for partial correlation, and Matplotlib/Seaborn for data visualization. Statistical significance for all tests was determined at an alpha level of *p* < 0.05.
