# Supplementary material for: A multi-scale framework for BOD5 prediction from water quality to hydro-geomorphic interpolation
Source: iScience. 2025 Nov 21;28(12):114177. doi: 10.1016/j.isci.2025.114177 (PMC12757620; doi:10.1016/j.isci.2025.114177)
Supplement: Document S1. Figure S1 and Tables S1–S5 [file mmc1.pdf]

iScience, Volume *28*

Supplemental information

A multi-scale framework for BOD_5 prediction from water quality to hydro-geomorphic interpolation

Amin Arzhangi and Sadegh Partani

Point Accuracy Metrics

1. **Coefficient of Determination (R^2):** Measures the proportion of the variance in the observed data that is predictable from the model.

   $$R^2 = 1 - [\Sigma (y_i - \hat{y}_i)^2 / \Sigma (y_i - \bar{y})^2] \quad \text{(Eq.S1)}$$

   Where $\bar{y}$ is the mean of the observed data.

2. **Mean Squared Error (MSE):** The average of the squares of the errors. It heavily penalizes large errors.

   $$MSE = (1/n) * \Sigma (y_i - \hat{y}_i)^2 \quad \text{(Eq.S2)}$$

3. **Root Mean Squared Error (RMSE):** The square root of the MSE, expressed in the same units as the predicted variable.

   $$RMSE = sqrt [(1/n) * \Sigma (y_i - \hat{y}_i)^2] \quad \text{(Eq.S3)}$$

4. **Mean Absolute Error (MAE):** The average of the absolute differences between prediction and actual observation.

   $$MAE = (1/n) * \Sigma|y_i - \hat{y}_i| \quad \text{(Eq.S4)}$$

Relative Error Metrics

1. **Mean Absolute Percentage Error (MAPE):** The mean of the absolute percentage errors.

   $$MAPE = (100/n) * \Sigma|(y_i - \hat{y}_i) / y_i| \quad \text{(Eq.S5)}$$

2. **Symmetric Mean Absolute Percentage Error (SMAPE):** A variation of MAPE that is less biased towards low forecast values.

   $$SMAPE = (100/n) * \Sigma [|\hat{y}_i - y_i| / (|y_i| + |\hat{y}_i|) / 2] \quad \text{(Eq.S6)}$$

3. **Mean Squared Logarithmic Error (MSLE):** Calculates the mean of the squared differences between the natural logarithm of the predicted and actual values.

$$MSLE = (1/n) * \Sigma (\log (1 + y\_i) - \log (1 + \hat{y}\_i))^2 \quad \text{(Eq.S7)}$$

__Uncertainty and Prediction Interval Metrics__

Let L_i and U_i be the lower and upper bounds of the 95% Prediction Interval for the *i*-th prediction, respectively.

1. **Prediction Interval Coverage Probability (PICP):** The percentage of observed values that fall within their 95% prediction intervals.

$$PICP = (1/n) * \Sigma c\_i \quad \text{(Eq.S8)}$$

where $c\_i = 1$ if $L\_i \leq y\_i \leq U\_i$, and $c\_i = 0$ otherwise.

2. **Mean Prediction Interval Width (MPIW):** The average width of the prediction intervals. A measure of precision.

$$MPIW = (1/n) * \Sigma (U\_i - L\_i) \quad \text{(Eq.S9)}$$

3. **Normalized Mean Prediction Interval Width (NMPIW):** The MPIW normalized by the range of the observed data.

$$NMPIW = MPIW / (y\_max - y\_min) \quad \text{(Eq.S10)}$$

4. **Coverage Width-based Criterion (CWC):** A composite metric that penalizes models with poor coverage (PICP < 95%).

$$CWC = MPIW * (1 + \gamma * \exp [-\eta (PICP - 0.95)]) \quad \text{(Eq.S11)}$$

Where $\gamma = 1$ if $PICP < 0.95$ and 0 otherwise, and η is a penalty parameter (e.g., 50).

Probabilistic Metrics

Let F_i be the predicted cumulative distribution function (CDF) for the *i*-th observation, and f_i be the predicted probability density function (PDF). For our Gaussian assumption, these are derived from N ($\hat{y}_i$, RMSE).

1. **Negative Log-Likelihood (NLL):** Measures the quality of the predicted probability distribution. Lower is better.

   $$NLL = - \Sigma \log(f_i(y_i)) \quad \text{(Eq.S12)}$$

2. **Continuous Ranked Probability Score (CRPS):** A comprehensive metric that generalizes the MAE to probabilistic forecasts.

   $$CRPS = (1/n) * \Sigma \int [F_i(x) - H (x - y_i)]^2 dx \quad \text{(Eq.S13)}$$

   where H(x) is the Heaviside step function.

Table S1 Calibrated Coefficients for the 2DPR Model without FC

Linear Terms		Quadratic Terms		Interaction Terms	
Predictor(s)	Coefficient (β)	Predictor(s)	Coefficient (β)	Predictor(s)	Coefficient (β)
T	-0.0587	T^2	+0.0310	T * Tu (NTU)	-0.0016
Tu (NTU)	-0.0089	Tu $(NTU)^2$	+0.0000	T * NO_3^- (mg/l)	-0.0426
NO_3^- (mg/l)	-1.9338	NO_3^- $(mg/l)^2$	+0.0014	T * DO (mg/l)	-0.0554
DO (mg/l)	+2.1414	DO $(mg/l)^2$	-0.1185	T * TS (mg/l)	-0.0017
TS (mg/l)	+0.0513	TS $(mg/l)^2$	+0.0000	T * FC (Colonies)	+0.0000
FC (Colonies)	-0.4506	FC $(Colonies)^2$	+0.0011	Tu (NTU) * NO_3^- (mg/l)	-0.0005
				Tu (NTU) * DO (mg/l)	+0.0003
				Tu (NTU) * TS (mg/l)	+0.0000
				Tu (NTU) * FC (Colonies)	+0.0010
				NO_3^- (mg/l) * DO (mg/l)	+0.3033
Intercept (β_0) = 35.85					

Table S2 Calibrated Coefficients for the 2DPR Model without NO_3^-

Linear Terms	Coeff. (β)	Quadratic Terms	Coeff. (β)	Interaction Terms	Coeff. (β)
T	+2.2307	T^2	-0.0635	T * Tu	-0.0022
Tu	+0.0718	Tu^2	-0.0000	T * DO	-0.0890
DO	+3.4116	DO^2	-0.2878	T * TS	+0.0007
TS	-0.0125	TS^2	+0.0000	T * FC	+0.0048
FC	-0.1264	FC^2	+0.0007	T * Total_P	-1.9712
Total_P	+152.1147	$Total\_P^2$	-253.9443	Tu * DO	-0.0039
				Tu * TS	-0.0000
				Tu * FC	+0.0004
				Tu * Total_P	+0.2721
				DO * TS	+0.0008
				DO * FC	-0.0038
				DO * Total_P	-12.4343
				TS * FC	-0.0001
				TS * Total_P	-0.0089
				FC * Total_P	-1.1293
Intercept (β_0) = -17.5383					

Table S3 Calibrated Coefficients for the 2DPR Model without FC and NO_3^-

Linear Terms	Coeff. (β)	Quadratic Terms	Coeff. (β)	Interaction Terms	Coeff. (β)
T	+3.1040	T^2	-0.0784	T * Tu	-0.0015
Tu	+0.0527	Tu^2	-0.0000	T * DO	-0.1477
DO	+4.9535	DO^2	-0.3705	T * TS	+0.0009
TS	-0.0163	TS^2	+0.0000	T * Total_P	-3.9786
Total_P	+189.2319	$Total\_P^2$	-347.1130	Tu * DO	-0.0026
				Tu * TS	+0.0000
				Tu * Total_P	+0.1332
				DO * TS	+0.0016
				DO * Total_P	-14.6500
				TS * Total_P	-0.0101
Intercept (β_0) = -28.7892					

Table S4 Results of the Shapiro-Wilk Normality Test on Cleaned Water Quality Parameters (N=63)

Parameter	W-Statistic	p-value	Verdict (α=0.05)
BOD_5 (mg/l)	0.725	< 0.001	Normal
DO (mg/l)	0.952	0.053	Non-normal
pH	0.827	< 0.001	Normal
T (°C)	0.932	0.008	Normal
NO_3^- (mg/l)	0.771	< 0.001	Normal
Total P (mg/l)	0.718	< 0.001	Normal
Tu (NTU)	0.584	< 0.001	Normal
TS (mg/l)	0.836	< 0.001	Normal
FC (Colonies)	0.470	< 0.001	Normal

Table S5 Asymmetric Matrix of Hydrologic Distances (km) and Sinuosity Indices (SI) Between Sampling Stations . The upper triangle (top-right) of the matrix displays the hydrologic distance (D_hydro) in kilometers, representing the actual path of water flow between stations. The lower triangle (bottom-left) displays the dimensionless Sinuosity Index (SI), calculated as the ratio of hydrologic distance to the direct Euclidean distance, quantifying the meandering nature of the reach.

	P1	P2	P3	P4	P5	P6	P7	P8	P9	P10	P11	P12	P13	P14	P15	P16	P17	P18	P19	P20
P1	-	18.32	34.26	41.69	79.12	86.81	67.59	71.39	58.69	70.79	64.39	91.72	84.32	91.45	95.84	103.27	114.11	133.00	139.82	140.73
P2	2.68	-	15.94	23.37	60.80	68.49	49.27	53.07	40.37	52.47	46.07	73.40	66.00	73.13	77.52	84.95	95.79	114.68	121.50	122.41
P3	2.69	1.25	-	9.63	47.06	54.75	35.53	39.33	26.63	38.73	32.33	59.66	52.26	59.39	63.78	71.21	82.05	100.94	107.76	108.67
P4	2.18	1.37	1.31	-	37.43	24.80	25.90	29.70	17.00	29.10	22.70	50.03	42.63	49.76	54.15	61.58	72.42	91.31	98.13	99.04
P5	2.77	2.22	2.96	3.59	-	24.47	36.08	36.88	24.18	12.08	29.88	57.21	49.81	56.94	61.33	68.76	79.60	98.49	105.31	106.22
P6	2.14	1.71	1.96	1.06	1.88	-	43.77	44.57	31.87	19.77	37.57	64.90	57.50	64.63	69.02	76.45	87.29	106.18	113.00	113.91
P7	2.76	2.47	2.29	2.79	2.45	1.60	-	24.60	11.90	24.00	17.60	44.93	37.53	44.66	49.05	56.48	67.32	86.21	93.03	93.94
P8	2.22	2.01	1.59	1.58	1.65	1.37	2.96	-	12.70	24.80	18.40	45.73	38.33	45.46	49.85	57.28	68.12	87.01	93.83	94.74
P9	1.81	1.43	1.23	1.18	1.82	1.42	2.25	1.24	-	8.35	5.70	33.03	25.63	32.76	37.15	44.58	55.42	74.31	81.13	82.04
P10	2.03	1.65	1.67	1.83	1.13	0.99	2.32	1.57	1.48	-	6.40	33.73	26.33	33.46	37.85	45.28	56.12	75.01	81.83	82.74
P11	1.71	1.36	1.21	1.17	1.91	1.60	1.45	1.24	1.14	1.43	-	31.45	19.93	27.06	31.45	38.88	49.72	68.61	75.43	63.81[1]
P12	1.81	1.53	1.55	1.58	2.46	2.24	1.78	1.68	1.91	2.49	2.26	-	7.40	14.53	18.92	26.35	37.19	63.48	62.90	63.81[1]
P13	1.65	1.40	1.32	1.32	1.94	1.85	1.46	1.42	1.43	1.62	1.35	1.15	-	7.13	11.52	18.95	29.79	48.68	55.50	56.41
P14	1.77	1.54	1.45	1.48	2.00	1.90	1.66	1.65	1.71	1.83	1.67	1.31	1.41	-	18.65	26.08	36.92	55.81	62.63	63.54
P15	1.55	1.35	1.26	1.25	1.66	1.66	1.33	1.32	1.29	1.38	1.21	0.96	1.00	1.99	-	7.43	18.27	37.16	43.98	44.89
P16	1.51	1.33	1.24	1.23	1.55	1.59	1.28	1.48	1.24	1.31	1.27	1.14	1.02	1.58	1.01	-	10.84	29.73	36.55	37.46
P17	1.46	1.30	1.22	1.21	1.47	1.53	1.25	1.41	1.21	1.27	1.22	1.16	1.05	1.39	1.07	1.08	-	18.89	25.71	26.62
P18	1.42	1.28	1.22	1.21	1.43	1.50	1.24	1.36	1.21	1.26	1.22	1.38	1.13	1.33	1.16	1.17	1.21	-	6.82	7.73
P19	1.42	1.29	1.24	1.23	1.43	1.50	1.26	1.37	1.23	1.28	1.24	1.24	1.16	1.34	1.20	1.21	1.28	1.45	-	0.91
P20	1.41	1.28	1.23	1.22	1.42	1.49	1.25	1.36	1.22	1.26	1.03	1.23	1.15	1.33	1.18	1.19	1.24	1.28	0.68[1]	-

- [1] Values of SI < 1 (e.g., between P19 and P20) or anomalous values (e.g., between P11, P12, and P20) are likely due to minor inaccuracies in GIS digitization or measurement over short, straight reaches and should be interpreted as SI ≈ 1.0, indicating a nearly straight channel.
- Blank diagonal indicates a distance/sinuosity of zero from a station to itself.

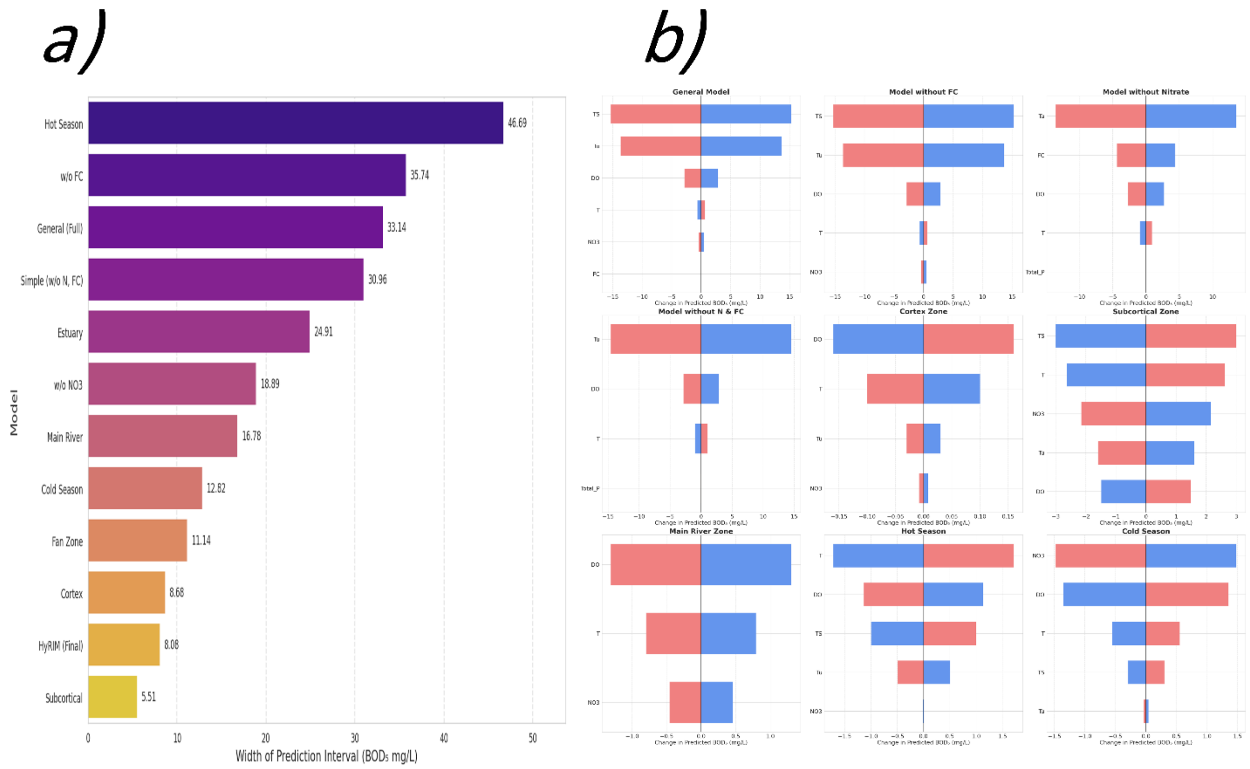

Fig.S1 a) Uncertainty of each model **b)** Sensitivity analysis
